# Serum concentration of eicosapentaenoic acid is associated with cognitive function in patients with coronary artery disease

**DOI:** 10.1186/1475-2891-13-112

**Published:** 2014-12-04

**Authors:** Shusuke Yagi, Tomoya Hara, Rie Ueno, Ken-ichi Aihara, Daiju Fukuda, Akira Takashima, Junko Hotchi, Takayuki Ise, Koji Yamaguchi, Takeshi Tobiume, Takashi Iwase, Hirotsugu Yamada, Takeshi Soeki, Tetsuzo Wakatsuki, Michio Shimabukuro, Masashi Akaike, Masataka Sata

**Affiliations:** Department of Cardiovascular Medicine, The University of Tokushima Graduate School of Health Biosciences, 3-18-15 Kuramoto-cho, Tokushima-city, Tokushima 770-8503 Japan; Department of Medicine and Bioregulatory Sciences, The University of Tokushima Graduate School of Health Biosciences, 3-18-15 Kuramoto-cho, Tokushima-city, Tokushima 770-8503 Japan; Department of Cardio-Diabetes Medicine, The University of Tokushima Graduate School of Health Biosciences, 3-18-15 Kuramoto-cho, Tokushima-city, Tokushima 770-8503 Japan; Department of Medical Education, The University of Tokushima Graduate School of Health Biosciences, 3-18-15 Kuramoto-cho, Tokushima-city, Tokushima 770-8503 Japan

**Keywords:** Eicosapentaenoic acid, n-3 polyunsaturated fatty acids, Cognitive function, Mini-mental state examinations, Coronary artery disease

## Abstract

**Background:**

Recent studies have shown that intake of n-3 polyunsaturated fatty acids (PUFAs) is associated with reduced risk of cognitive impairment and coronary artery disease (CAD); however, it is currently unknown whether reduced serum n-3 PUFA is associated with cognitive impairment in patients with CAD.

**Methods:**

We retrospectively evaluated cognitive function with the mini-mental state examination (MMSE), serum levels of PUFAs (including eicosapentaenoic acid [EPA], docosahexaenoic acid [DHA], dihomogammalinolenic acid [DGLA], and arachidonic acid [AA]), cardiovascular risk factors (hypertension, dyslipidemia, diabetes mellitus, cerebrovascular disease, and history of current/previous smoking), and parameters of cardiac function (left ventricular ejection fraction and brain natriuretic peptide levels) in 146 Japanese CAD patients. The associations between the MMSE scores and the other parameters were evaluated.

**Results:**

Pearson correlation analysis showed that EPA (R = 0.25, P <0.01), EPA/AA ratio (R = 0.22, P = 0.01), and left ventricular ejection fraction (R = 0.15, P = 0.04) were positively associated with MMSE score, and that age (R = −0.20, P <0.01) and brain natriuretic peptide levels (R = −0.28, P <0.01) were inversely associated with MMSE score. Multiple regression analysis showed that age (P <0.05) was negatively associated with MMSE score, while EPA (P <0.01) and EPA/AA ratio (P <0.05) were positively associated with MMSE score; however, sex; body mass index; left ventricular ejection fraction; levels of DHA, AA, and DGLA; DHA/AA ratio; brain natriuretic peptide; and presence of hypertension, dyslipidemia, diabetes mellitus, cerebrovascular disease, and history of current/previous smoking were statistically excluded.

**Conclusions:**

Serum EPA concentration is associated with cognitive function in patients with CAD, suggesting that a low serum EPA level is a risk factor for cognitive impairment independent of cardiac function, including left ventricular ejection fraction. This correlation potentially lends further support to a role of dietary n-3 PUFAs in preventing the cognitive decline in CAD patients.

**Electronic supplementary material:**

The online version of this article (doi:10.1186/1475-2891-13-112) contains supplementary material, which is available to authorized users.

## Background

Cardiovascular disease has recently been implicated as a major factor in the development of dementia, as these diseases may be linked by shared common risks and pathogenic elements [1, 2]. Accumulation of cardiovascular risk factors therefore leads to cognitive impairment. In addition, hypoxia/ischemia resulting from reduced cerebral blood flow due to cardiac dysfunction may be associated with dementia. Conversely, dementia itself could be an independent cardiovascular risk factor; patients with dementia have lifestyle-related problems, such as inappropriate food or alcohol intake, sedentary activity, and psychosocial stress, including depression [3]. Therefore, the modification of these lifestyle-related problems could be strategies for coronary artery disease (CAD) and dementia prevention. While several pharmacological agents, including cholinesterase inhibitors [4], have been developed for dementia, sufficiently effective and curative treatments have not yet been established. Therefore, the identification of residual risk factors is important for dementia prevention.

The Japan Eicosapentaenoic Acid Lipid Intervention Study showed that long-term use of eicosapentaenoic acid (EPA) is effective for prevention of major coronary events in Japanese hypercholesterolemic patients [5]. In addition, recent studies demonstrated that consumption of fish and n-3 polyunsaturated fatty acids (PUFAs) reduced the incidence of cognitive impairment [6]. These studies indicate that a reduced serum level of n-3 PUFAs may be a risk factor for both CAD and cognitive impairment.

However, it is currently unknown whether reduced serum levels of n-3 PUFAs are associated with cognitive impairment, and more specifically, which components of PUFAs are associated with cognitive function in CAD patients. Therefore, the aim of this study was to investigate the association between cognitive function and n-3 PUFA levels (including eicosapentaenoic acid [EPA], docosahexaenoic acid [DHA], dihomogammalinolenic acid [DGLA], and arachidonic acid [AA]) in CAD patients, and to identify which components of PUFAs are associated with cognitive function in these patients.

We hypothesized that decreased level of EPA would be associated with cognitive impairment in patients with CAD.

## Material and methods

### Patients and study design

In patients with CAD, serum PUFA levels were measured for identification of residual risk factors for CAD. In addition, patients underwent mini-mental state examinations (MMSE) to screen cognitive function [1]. We retrospectively reviewed 146 consecutive Japanese patients diagnosed with CAD in the Department of Cardiology at Tokushima University Hospital between April 2013 and March 2014.

Patients with CAD were defined as patients with a history of myocardial infarction, angiographic evidence of at least 50% stenosis by area in at least 1 coronary artery, evidence of exercise-induced ischemia, or history of coronary revascularization. The exclusion criteria were as follows: use of fish oil supplements or n-3 fatty acid-containing drugs or a history of myocardial infarction within 1 month. In addition, patients with symptomatic active malignant diseases or liver dysfunction (aspartate aminotransferase levels >100 IU/L, alanine aminotransferase levels >100 IU/L) were also excluded.

Hypertensive patients were defined as those with a systolic blood pressure of ≥140 mmHg and/or diastolic blood pressure of ≥90 mmHg and/or individuals receiving antihypertensive medications. Dyslipidemic patients were defined as those with a low-density lipoprotein cholesterol level (LDL-C) of ≥140 mg/dL, a triglyceride level of ≥150 mg/dL, a high-density lipoprotein cholesterol level (HDL-C) of <40 mg/dL, or individuals receiving lipid-lowering medications. Diabetic patients were defined as individuals receiving insulin or oral hypoglycemic agents or those with an HbA1c level of ≥6.5%, fasting plasma glucose level of 126 mg/dL, or non-fasting plasma glucose level of ≥200 mg/dL.

Serum fatty acid composition, including levels of EPA, DHA, DGLA, and AA, was measured by gas–liquid chromatography at a commercially available laboratory (SRL, Tokyo, Japan).

Since n-6 PUFAs, including AA, are often considered to be pro-inflammatory fatty acids and the EPA/AA ratio is associated with a low incidence of cardiac events [7], EPA/AA and DHA/AA ratios were also calculated.

In addition, other biochemical parameters, including LDL-C, HDL-C, triglycerides, fasting plasma glucose, and HbA1c, were also measured.

Cardiac function was evaluated by measuring brain natriuretic peptide (BNP), a biomarker which represents left ventricular endodiastolic pressure, and left ventricular ejection fraction (LVEF) evaluated by echocardiography. The LVEF was calculated using the modified Simpson's method with the apical 4-chamber and 2-chamber views.

Cognitive function was evaluated by MMSE, which is widely used as a screening tool for assessment of cognitive function [8].

This study protocol was approved by the Tokushima University Hospital Ethics Committee.

### Statistical analysis

Continuous variables were averaged and expressed as the mean ± standard deviation, and categorical parameters were expressed as a percentage. MMSE scores and BNP levels were natural log transformed for statistical analysis because of non-normal distributions. Associations between CAD risk factors and MMSE scores were determined by the Student’s t-test or the Pearson’s correlation analysis. Multiple regression analysis (standard least squares method) was used to assess the degrees of association between the CAD risk factor variables and the MMSE scores. Residuals and residual vs. fit plots were examined to ensure homoscedasticity.

All statistical analyses were performed using JMP 10 software. Statistical significance was defined as P <0.05.

## Results

### Clinical characteristics of subjects

**Figure 1:**
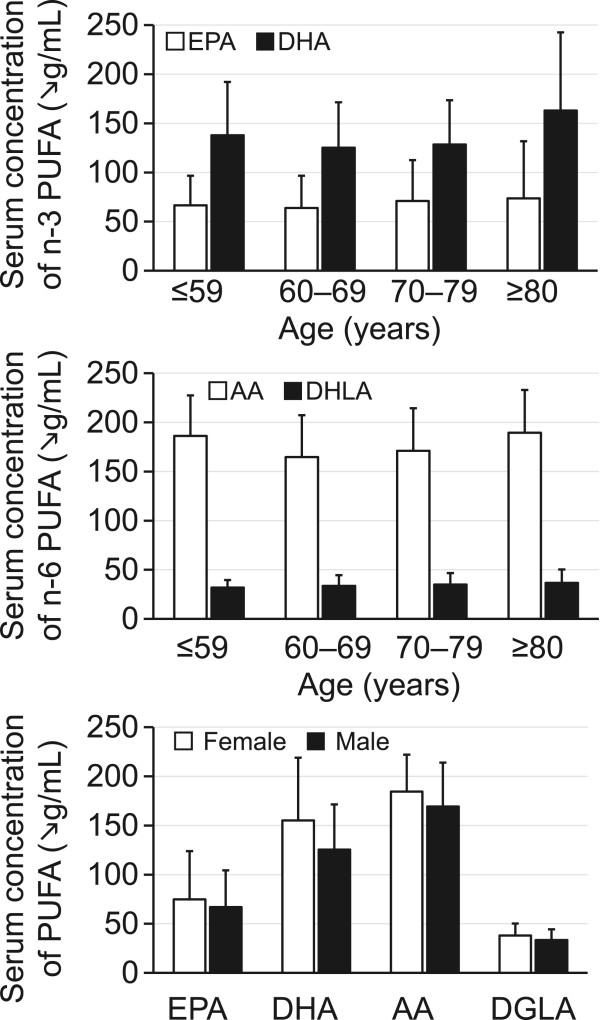
**Distribution of levels of n-3 and n-6 polyunsaturated fatty acids (PUFAs) stratified by age and sex.** EPA, eicosapentaenoic acid; DHA, docosahexaenoic acid; AA, arachidonic acid; DGLA, dihomogammalinolenic acid.

Patient characteristics are shown in Table 1. The mean MMSE score was 27.8 ± 3.1, indicating that subjects generally had mild cognitive impairment. The serum concentration of n-3 PUFAs, including EPA and DHA, and n-6 PUFAs, including AA and DGLA, are shown in Figure 1 by patient age and sex. EPA and DHA concentrations were the highest among patients aged ≥80 years, and levels of EPA, DHA, DGLA, and AA were higher in women than in men.

**Table 1:** **Clinical characteristics of subjects**

Variables	
Number of patients	146
Mini-mental state examination score	27.8 ± 3.1
Male, n (%)	111 (76.0%)
Age, y	70.9 ± 8.6
Body mass index, kg/m^2^	23.5 ± 3.4
Systolic blood pressure, mmHg	127 ± 16.3
Diastolic blood pressure, mmHg	71 ± 11.6
Low-density lipoprotein cholesterol, mg/dL	96.0 ± 34.4
Triglycerides, mg/dL	124.5 ± 70.3
High-density lipoprotein cholesterol, mg/dL	55.0 ± 14.7
HbA1c, %	6.7 ± 5.5
Fasting plasma blood glucose, mg/dL	130.0 ± 58.7
Brain natriuretic peptide, pg/mL	151.6 ± 274.2
Left ventricular ejection fraction, %	58.6 ± 11.2
Fatty acids	
Docosahexaenoic acid, μg/mL	132.5 ± 52.2
Eicosapentaenoic acid, μg/mL	68.9 ± 40.4
Arachidonic acid, μg/mL	173.0 ± 43.4
Dihomogammalinolenic acid, μg/mL	34.5 ± 11.4
Complications	
Dyslipidemia, n (%)	126 (86.3%)
Hypertension, n (%)	131 (89.7%)
Diabetes mellitus, n (%)	80 (54.8%)
Cerebrovascular disease, n (%)	25 (17.1%)
Smoking, n (%)	108 (74.0%)
Drugs	
ACEI/ARB, n (%)	91 (62.3%)
β-blockers, n (%)	53 (36.3%)
Calcium channel blockers, n (%)	62 (42.5%)
Statins, n (%)	123 (84.2%)
Ezetimibe, n (%)	8 (5.5%)
Diuretics loop, n (%)	27 (18.5%)
Mineralocorticoid antagonists, n (%)	7 (4.8%)

Unless indicated otherwise, data are presented as the mean ± standard deviation.

ACEI, angiotensin converting enzyme inhibitors; ARB, angiotensin II receptor blockers.

### Correlation between n-3 PUFAs and MMSE score

**Figure 2:**
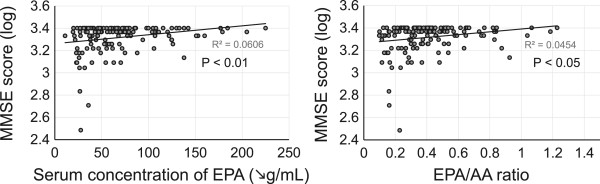
**Association between serum concentration of eicosapentaenoic acid (EPA) and EPA/arachidonic acid (AA) ratio and mini-mental state examination (MMSE) score.**

**Figure 3:**
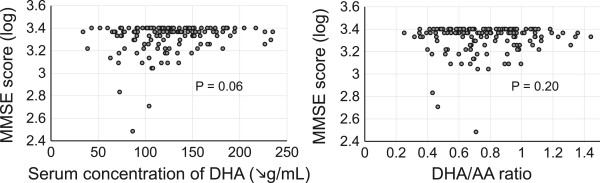
**There were no associations between serum concentration of docosahexaenoic acid (DHA) and DHA/AA ratio and mini-mental state examination (MMSE) score.**

The Pearson correlation analysis showed that levels of EPA, EPA/AA ratio, and LVEF were positively associated with MMSE score, and that age and BNP was inversely associated with MMSE score (Table 2, Figure 2). There were no relationships between MMSE score and body mass index or levels of DHA, AA, DGLA, and DHA/AA ratio (Figure 3). In addition, there were no differences in MMSE scores according to patient sex and risk factors, including hypertension, dyslipidemia, diabetes mellitus, cerebrovascular disease, and history of current/previous smoking. Interestingly, both LVEF and BNP (parameters of cardiac function) were associated with serum levels of DHA and DHA/AA but not EPA or EPA/AA (Additional file 1: Tables S1 and S2).

**Table 2:** **Pearson correlation analysis between MMSE scores and cardiovascular risk factors**

Variables	R	P-value
Age	−0.20	< 0.01
Sex	-	0.30
Body mass index	0.09	0.23
DHA	0.12	0.06
EPA	0.25	<0.01
AA	0.1	0.99
DGLA	−0.001	0.20
EPA/AA	0.22	0.01
DHA/AA	0.10	0.20
Left ventricular ejection fraction	0.15	0.04
Brain natriuretic peptide	−0.28	< 0.01
Hypertension	-	0.96
Dyslipidemia	-	0.89
Diabetes mellitus	-	0.37
Cerebrovascular disease	-	0.79
Current/previous smoker	-	0.12

MMSE, mini-mental state examination; DHA, docosahexaenoic acid; EPA, eicosapentaenoic acid; AA, arachidonic acid; DGLA, dihomogammalinolenic acid.

Multiple regression analysis was then performed to elucidate independent determinants of MMSE score; age (P <0.05) was negatively associated with MMSE score, while EPA levels (P <0.01) and EPA/AA ratio (P <0.05) were positively associated with MMSE score. However, sex; body mass index; levels of DHA, AA, DGLA, and DHA/AA ratio; LVEF; levels of BNP; presence of hypertension, dyslipidemia, diabetes mellitus, and cerebrovascular disease; and history of current/previous smoking were statistically excluded (Table 3).

**Table 3:** **Multiple regression analysis for determinants of degree of MMSE score in patients with CAD**

Variables	Coefficient	95% confidence interval	P-value
**Model 1**			
Age, years	−0.004	−0.007 to −0.001	0.02
Male sex	0.005	−0.03 to 0.04	0.76
Body mass index, kg/m^2^	−0.0008	−0.001 to 0.007	0.85
Hypertension	0.02	−0.01 to 0.06	0.24
Dyslipidemia	−0.001	−0.04 to 0.03	0.91
Diabetes mellitus	0.001	−0.02 to 0.02	0.98
Cerebrovascular disease	−0.01	−0.04 to 0.02	0.50
Current/previous smoker	0.001	−0.03 to 0.04	0.80
EPA, μg/mL	0.001	0.0003 to 0.002	< 0.01
DHA, μg/mL	−0.0004	−0.001 to 0.0003	0.22
AA, μg/mL	0.0001	−0.0001 to 0.001	0.83
DGLA, μg/mL	0.001	−0.002 to 0.004	0.45
LVEF, %	0.001	−0.001 to 0.004	0.20
BNP, pg/mL	−0.015	−0.035 to 0.005	0.15
**Model 2**			
Age, years	−0.003	−0.006 to −0.0002	0.04
Male sex	0.002	−0.03 to 0.04	0.89
Body mass index, kg/m^2^	0.0008	−0.006 to 0.008	0.83
Hypertension	0.02	−0.02 to 0.05	0.37
Dyslipidemia	0.003	−0.03 to 0.04	0.84
Diabetes mellitus	0.002	−0.03 to 0.04	0.85
Cerebrovascular disease	−0.01	−0.03 to 0.02	0.58
Current/previous smoker	0.003	−0.03 to 0.04	0.85
EPA/AA	0.15	0.03 to 0.28	0.01
DHA/AA	−0.04	−0.15 to 0.07	0.42
LVEF, %	0.001	−0.001 to 0.003	0.29
BNP, pg/mL	−0.018	−0.04 to 0.001	0.06

Model 1 R^2^ = 0.20; P <0.05.

Model 2 R^2^ = 0.17; P <0.05.

EPA, eicosapentaenoic acid; DHA, docosahexaenoic acid; AA, arachidonic acid; DGLA, dihomogammalinolenic acid; LVEF, left ventricular ejection fraction; BNP, brain natriuretic peptide.

## Discussion

The present study demonstrated that decreased serum levels of EPA and a reduced EPA/AA ratio are associated with cognitive impairment in patients with CAD, indicating that decreased EPA is a risk factor for development of cognitive impairment this patient population. AA levels showed no association with MMSE scores, while low EPA levels were independently correlated with MMSE scores. Therefore, EPA/AA did not increase the predictive power of EPA alone. In addition, the Pearson correlation analysis exhibited an association between MMSE scores and LVEF or BNP; however, multiple regression in an age-adjusted model showed no association. Although there is a possibility of confounding between cardiac function and age, our results indicate that MMSE scores were associated with EPA levels independent of cardiac function.

It has been reported that fish intake is associated with cognitive function [9, 10], and n-3 PUFA-containing food improves cognitive function [11]. The exact mechanisms of EPA on neuronal function are unknown; however, EPA is involved in endothelial-dependent vascular function and plays important roles in the prevention of microcirculatory insufficiency. Therefore, one possible mechanism is that decreased EPA results in microcirculation insufficiency in the brain, thus leading to ischemia-induced brain damage. It has been reported that EPA enhances endothelial expression of nitric oxide synthase, and activation of endothelial nitric oxide synthase leads to vasodilation and endothelial protection [12, 13]. Reduced nitric oxide synthase-induced microcirculation insufficiency may be involved in the development of cognitive impairment. Brain microvascular insufficiency, including cerebral white matter disease, is an early marker of cognitive impairment [14, 15]. Thus, in patients with very mild cognitive impairment, such as those who were included in this study, microcirculation insufficiency rather than amyloid β deposition, the cause of Alzheimer’s disease, may be the predominant contributor to vascular dementia.

It has been reported that vascular inflammation is associated with cognitive function [16]. EPA is known to have the ability to attenuate tumor necrosis factor-α-induced upregulation of vascular cell adhesion molecule-1, intercellular adhesion molecule-1, and monocyte chemoattractant protein-1 [17]. The anti-inflammatory effects of EPA might contribute to the prevention of cognitive impairment.

A previous meta-analysis demonstrated that subjects with predementia syndrome had significantly lower levels of EPA but not DHA or total n-3 PUFA [18]; thus, serum EPA may be a more sensitive biomarker for prediction of cognitive impairment compared to serum DHA. It has also been reported that administration of n-3 PUFA to patients with very mild Alzheimer’s disease (MMSE >27 points) delayed the rate of cognitive dysfunction, indicating the importance of n-3 PUFA on brain function in early stages of cognitive impairment [19].

In addition, EPA is converted into DHA in the liver and at the blood-brain barrier and has been shown to cross the blood-brain barrier after dietary supplementation, thereby suggesting that EPA plays important roles in cognitive function after conversion into DHA [20, 21]. EPA could be a rich source of DHA and may compensate for reduced levels of DHA in the brain; decreased serum EPA may thus be a more profound indicator of cognitive function than decreased serum DHA.

The present study had several limitations. In particular, this was a retrospective study with a small sample size in a single center. Larger clinical cohort studies are needed to clarify the effects of EPA on cognitive function.

## Conclusions

Serum EPA concentration rather than serum DHA concentration is associated with cognitive function in patients with CAD, suggesting that a low serum EPA level is a risk factor for cognitive impairment independent of cardiac function. Our data support a role of dietary n-3 PUFAs in preventing the cognitive decline in CAD patients.

## Electronic supplementary material

Additional file 1: Table S1: Pearson correlation analysis between left ventricular ejection fraction and serum levels of polyunsaturated fatty acids. **Table S2.** Pearson correlation analysis between brain natriuretic peptide levels and serum levels of polyunsaturated fatty acids. (DOCX 16 KB)

## Abbreviations

PUFAs: Polyunsaturated fatty acids
EPA: Eicosapentaenoic acid
DHA: Docosahexaenoic acid
DGLA: Dihomogammalinolenic acid
AA: Arachidonic acid
CAD: Coronary artery disease
MMSE: Mini-mental state examinations
LDL-C: Low-density lipoprotein cholesterol level
HDL-C: High-density lipoprotein cholesterol level.
