# Supplementary material for: Serum concentration of eicosapentaenoic acid is associated with cognitive function in patients with coronary artery disease
Source: Nutr J. 2014 Dec 4;13:112. doi: 10.1186/1475-2891-13-112 (PMC4391466; doi:10.1186/1475-2891-13-112)
Supplement: Supplementary file 1 — Additional file 1: Table S1: Pearson correlation analysis between left ventricular ejection fraction and serum levels of polyunsaturated fatty acids. Table S2. Pearson correlation analysis between brain natriuretic peptide levels and serum levels of polyunsaturated fatty acids. (DOCX 16 KB) [file 12937_2014_878_MOESM1_ESM.docx]

**Additional file 1: Table S1**

Pearson correlation analysis between left ventricular ejection fraction and serum levels of polyunsaturated fatty acids

| Variables | R | P-value |
| --- | --- | --- |
| DHA | 0.23 | <0.01 |
| EPA | 0.10 | 0.22 |
| AA | 0.08 | 0.35 |
| DGLA | 0.10 | 0.24 |
| EPA/AA | 0.06 | 0.45 |
| DHA/AA | 0.22 | <0.01 |

AA, arachidonic acid; DGLA, dihomogammalinolenic acid; DHA, docosahexaenoic acid; EPA, eicosapentaenoic acid

**Supplemental Table 2**

Pearson correlation analysis between brain natriuretic peptide levels and serum levels of polyunsaturated fatty acids

| Variables | R | P-value |
| --- | --- | --- |
| DHA | −0.25 | <0.01 |
| EPA | −0.08 | 0.31 |
| AA | −0.14 | 0.07 |
| DGLA | −0.22 | <0.01 |
| EPA/AA | −0.02 | 0.77 |
| DHA/AA | −0.24 | <0.01 |

AA, arachidonic acid; DGLA, dihomogammalinolenic acid; DHA, docosahexaenoic acid; EPA, eicosapentaenoic acid
